# Genomic Epidemiology of NDM-1 Carbapenemase-Producing *Acinetobacter* spp. from Hospital Wastewater in Shenzhen, China

**DOI:** 10.3390/antibiotics15040347

**Published:** 2026-03-27

**Authors:** Xiaoqian Guo, Yulin Fu, Xinxin Chen, Yiying Cheng, Huimin Li, Dalin Hu, Suli Huang, Liangqiang Lin, Ziquan Lv

**Affiliations:** 1 School of Public Health, Southern Medical University, Guangzhou 510515, China; xq1214201@163.com (X.G.); cyy0066882024@163.com (Y.C.); smuhdl@126.com (D.H.); 2 Division of Conservation and Application of Biological Resources, Shenzhen Center for Disease Control and Prevention, Shenzhen 518055, China; fyl199287@163.com (Y.F.); lihuimin0203@126.com (H.L.); 3 School of Medicine, Southern University of Science and Technology, Shenzhen 518055, China; 12333058@mail.sustech.edu.cn; 4 School of Public Health, Shenzhen University Medical School, Shenzhen University, Shenzhen 518055, China; grace420@szu.edu.cn

**Keywords:** *Acinetobacter*, hospital wastewater, antimicrobial resistance, carbapenem resistance, plasmids, *bla*_NDM_

## Abstract

**Background:** Hospital wastewater (HWW) is a critical reservoir for carbapenem-resistant Gram-negative bacteria. **Methods:** Between November 2024 and August 2025, sixty 24 h composite wastewater samples were collected from five tertiary hospitals. Of the 244 carbapenem-resistant isolates recovered, 34 *bla*_NDM-1_-positive *Acinetobacter* isolates were subjected to phenotypic, genotypic, and plasmid analyses. **Results:** Eleven species were identified among the 34 carbapenem-resistant *Acinetobacter* isolates, predominantly non-*baumannii Acinetobacter* (NBA). All isolates were carbapenem-resistant (34/34, 100%) with high-level MICs (meropenem MIC_50/90_, 32/64 mg/L; imipenem MIC_50/90_, >128/>128 mg/L); 21% (7/34) of isolates were resistant to colistin, and resistance to ceftazidime, cefepime, and trimethoprim-sulfamethoxazole was 100%, 94%, and 76%, respectively. Core-genome SNP analysis revealed highly similar isolates across hospitals within the same season (1-2 SNPs) or within the same hospital across seasons (19 SNPs). Genomic analysis showed that *bla*_NDM-1_ was present in all isolates (34/34, 100%), with plasmid carriage in 85.3% (29/34); *bla*_OXA-58_ co-occurred in 62.1% (18/29), mainly on Rep_3 plasmids (19/29), especially R3-T28 (15/29) that frequently carried *bla*_OXA-58_ (10/15). Two unclassified plasmids co-harboring *bla*_NDM-1_ and *bla*_OXA-23_ were detected in *Acinetobacter tandoii* isolates. The *bla*_NDM-1_ gene was embedded in a conserved Tn*125*-like structures with variable flanks. **Conclusions:** Overall, carbapenem-resistant *Acinetobacter* from hospital wastewater frequently carried Rep_3 plasmid-borne *bla*_NDM-1_, especially R3-T28 and often co-occurring with *bla*_OXA-58_, within a conserved Tn*125*-like core structures. These findings highlight HWW as a potential hotspot for dissemination of carbapenem resistance and support routine genomic surveillance under a One Health framework.

## 1. Introduction

Antimicrobial resistance (AMR) represents a major global health threat, demanding coordinated surveillance across clinical, animal, and environmental sectors under a “One Health” framework [1]. Among various environmental niches, hospital wastewater (HWW) has been identified as a critical hotspot for the selection and dissemination of antibiotic-resistant bacteria (ARB) and antibiotic resistance genes (ARGs) [2]. HWW typically contains a complex mixture of antimicrobial residues, disinfectants, and diverse microbial populations derived from clinical activities. These conditions create intense selective pressure, fostering the persistence of ARB and accelerating horizontal gene transfer (HGT) mediated by mobile genetic elements (MGEs) such as plasmids, transposons, and integrons [3]. Numerous studies have documented significantly higher abundances of clinically relevant ARGs in HWW compared to municipal wastewater, highlighting the role of hospital effluents as important reservoirs and dissemination pathways for antimicrobial resistance [3,4,5].

Carbapenems are often considered the “last-resort” antibiotics for treating severe infections caused by multidrug-resistant (MDR) Gram-negative pathogens [6]. However, the emergence and global spread of carbapenemase-producing bacteria have severely compromised the clinical utility of carbapenems and are now prioritized in international AMR surveillance [7,8]. Major carbapenemase genes are frequently associated with mobile genetic elements: *bla*_NDM_ has disseminated worldwide across diverse Gram-negative hosts and is commonly linked to plasmids and transposon-rich platforms, such as Tn*125*/IS*Aba125*-related structures, facilitating efficient horizontal spread [9,10]. In parallel, *bla*_OXA_ carbapenemases have expanded in two major contexts—OXA-48-like enzymes are widely disseminated in *Enterobacterales* [11], whereas acquired OXA-type carbapenemases (notably OXA-23-like, OXA-24/40-like, and OXA-58-like) are key contributors to carbapenem resistance in *Acinetobacter* spp. [12]. Collectively, these trends highlight the urgency of tracking carbapenemase genes and their mobile vehicles across clinical-environment interfaces, including hospital wastewater [2].

During systematic surveillance of carbapenem-resistant bacteria in HWW, we observed a striking phenomenon: among the various bacterial genera isolated, *Acinetobacter* was the only genus in which all isolates carried *bla*_NDM-1_. This finding prompted us to focus on *Acinetobacter* as a key bacterial group potentially driving the persistence and spread of NDM-type carbapenemases in the HWW environment.

The genus *Acinetobacter* comprises Gram-negative, non-fermentative coccobacillary bacteria that are ubiquitous in both healthcare settings and natural ecosystems [13]. According to the List of Prokaryotic names with Standing in Nomenclature (LPSN) taxonomy database, the genus *Acinetobacter* comprised 90 child taxa with validly published and correct names at the time of access (20 November 2025) [14], reflecting substantial taxonomic expansion and ecological diversity. While *A. baumannii* is a notorious nosocomial pathogen renowned for its ability to acquire multidrug resistance [15], non-*baumannii Acinetobacter* (NBA) species—including *A. pittii*, *A. nosocomialis*, *A. lwoffii*, and *A. bereziniae*, among others—are increasingly recognized as opportunistic pathogens capable of harboring clinically significant resistance determinants [16]. Carbapenem-resistant Acinetobacter, including NBA species, have been repeatedly detected in HWW in multiple regions [17,18,19]. The most clinically important carbapenemases in *Acinetobacter* are the New Delhi metallo-β-lactamases (NDM) and the class D oxacillinases (OXA-type), particularly OXA-23 and OXA-58 [12,20]. While OXA-type enzymes often require upstream insertion sequences (e.g., IS*Aba1* or IS*Aba3*) for strong expression [21,22], the *bla*_NDM_ gene is usually embedded in highly mobile genetic platforms, conferring a strong dissemination potential [23].

In China, *bla*_NDM-1_ has been identified in various *Acinetobacter* species from clinical, environmental, and HWW sources [24,25,26,27]. Despite the growing clinical relevance of NBA species [28], research has remained heavily skewed toward clinical isolates. Systematic genomic analyses of NBA from HWW—a critical but understudied AMR reservoir—remain limited. Our study provides a comprehensive genomic characterization of *Acinetobacter* spp. carrying *bla*_NDM-1_ and OXA-type carbapenemase genes (e.g., *bla*_OXA-58_/*bla*_OXA-23_) isolated from wastewater from five tertiary hospitals in Shenzhen, China. Our findings reveal the species diversity, carbapenemase gene contexts, and plasmid-associated genetic platforms present in HWW, offering important insights into the environmental persistence and genomic plasticity of carbapenem-resistant *Acinetobacter*.

## 2. Results

### 2.1. Species Distribution and Antimicrobial Susceptibility Profiles

To maximize the diversity of carbapenem-resistant bacteria, a total of 60 hospital wastewater samples were cultured on BHI agar and on CHROMagar™ Orientation plates both supplemented with meropenem (2 µg/mL), yielding a total of 319 bacterial isolates in total from pre-disinfection hospital wastewater. Antimicrobial susceptibility testing (AST) showed that 244/319 (76.5%) isolates were resistant to meropenem. In parallel, PCR screening identified 100 *bla*_NDM_-positive isolates; the predominant groups were *Acinetobacter* spp. (34%), *Enterobacter* spp. (18%), and *Klebsiella* spp. (15%) (Figure S1). Notably, no carbapenem-resistant isolates were recovered from post-disinfection effluents.

**Figure 1:**
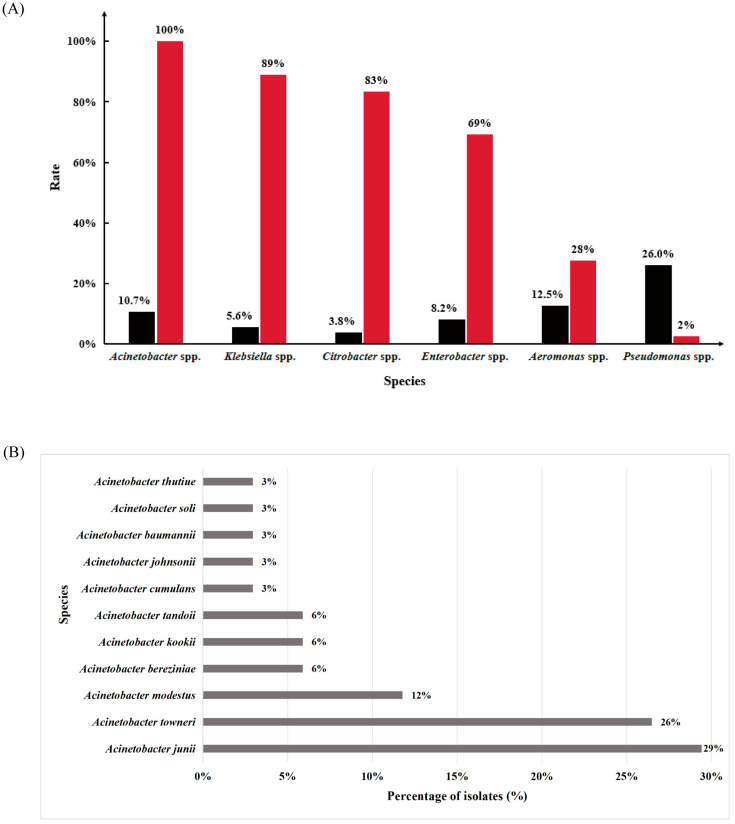
(**A**) Comparison of *bla*_NDM-1_ detection rates among different bacterial genera. Black bars represent the proportion of each genus among the 319 isolates, while red bars indicate the percentage of *bla*_NDM-1_-positive strains within each genus. (**B**) Species composition and distribution of 34 carbapenem-resistant *Acinetobacter* isolates. Final ANI-based species assignments are summarized in Table S1. In panel (**B**), isolates that did not reach the 95% ANI species-level cutoff were retained within the corresponding *A. towneri* or *A. modestus* visualization groups, and their final ANI-based assignments are provided in Table S1.

Among the *bla*_NDM_-positive isolates, 34 *Acinetobacter* isolates were recovered and subjected to further analyses. All 34 *Acinetobacter* isolates were resistant to carbapenems (34/34, 100%; Table 1) and carried *bla*_NDM-1_ (34/34, 100%; Figure 1A). Based on the visualization grouping shown in Figure 1B, these 34 carbapenem-resistant *Acinetobacter* isolates were distributed across 11 species-level categories. *A. junii* was the most prevalent group (10/34, 29%), followed by the *A. towneri* group (9/34, 26%). Other groups included the *A. modestus* group (4/34, 12%), *A. bereziniae*, *A. kookii* and *A. tandoii* (each 2/34, 6%), while *A. cumulans*, *A. johnsonii*, *A. baumannii*, *A. soli*, and *A. thutiue* were each represented by a single isolate (1/34, 3% each). Whole-genome average nucleotide identity (ANI) analysis further refined species assignments (Table S1). Species-level assignment was considered supported at ANI ≥ 95%. Under this criterion, 27/34 isolates were confidently assigned to the species level, including one isolate reassigned from *Acinetobacter* spp. to *A. thutiue*. The remaining seven isolates did not reach the ANI cutoff and were conservatively treated as unresolved at the species level.

AST demonstrated a multidrug-resistant phenotype among the *bla*_NDM-1_-positive *Acinetobacter* isolates (Table 1 and Table S2). Resistance rates reached 100% for meropenem, imipenem, and ceftazidime, while high resistance rates were also observed for cefepime (94%), trimethoprim-sulfamethoxazole (76%), gentamicin (68%), and ciprofloxacin (67%). In contrast, lower resistance rates were observed for tigecycline (0%), doxycycline (3%), amikacin (9%), tetracycline (47%) and piperacillin-tazobactam (53%). Tigecycline susceptibility was interpreted using FDA *Enterobacterales* breakpoints. Colistin MICs were determined by broth microdilution and interpreted for *Acinetobacter* spp., under which 21% of isolates were categorized as resistant. High minimum inhibitory concentrations (MICs) for meropenem and imipenem indicated high-level carbapenem resistance (MIC_50_/MIC_90_: 32/64 mg/L and >128/>128 mg/L, respectively).

### 2.2. Phylogenomic Relationships and Antimicrobial Resistance Gene Profiles

**Figure 2:**
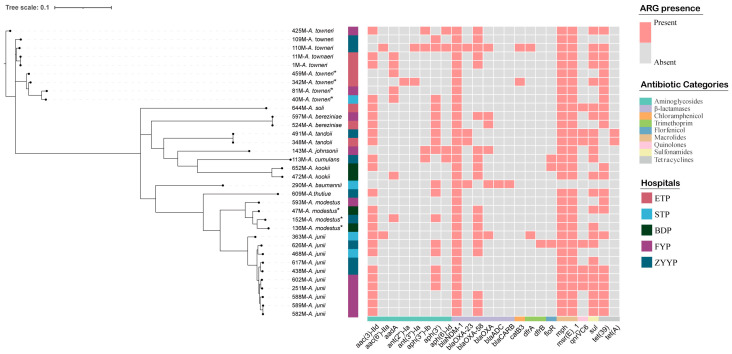
Phylogenetic relationships and ARG profiles of 34 carbapenem-resistant *Acinetobacter* isolates. The tree on the left shows the clustering relationships among isolates, and the heatmap on the right shows ARG distribution. Pink squares indicate presence of the corresponding ARGs, whereas gray squares indicate absence. The colored bars denote hospital source. Asterisks indicate isolates unresolved at the species level under the 95% ANI cutoff and labeled by their closest reference species.

Phylogenetic tree and core-genome SNP analysis revealed several highly related isolate pairs differing by only 0–20 SNPs, including pairs recovered within the same season from the same or different hospitals: 1M vs. 11M (*A. towneri*, ETP, 2024.11; 0 SNP), 348M vs. 491M (*A. tandoii*, ETP vs. ZYYP, 2025.05; 1 SNP), and 524M vs. 597M (*A. bereziniae*, ETP vs. FYP, 2025.08; 2 SNPs). A closely related pair was also observed within the same hospital across seasons (251M vs. 602M, *A. junii*, FYP, between March and September 2025; 19 SNPs) (Figure 2; Table S3).

In the present study, the *bla*_NDM-1_ gene was detected in all isolates (*n* = 34), while additional carbapenemase genes were variably detected, including *bla*_OXA-58_ (*n* = 19) and *bla*_OXA-23_ (*n* = 4). Besides carbapenemases, several ARGs also showed high detection rates (≥50%), notably *mph*(E) (*n* = 33), *msr*(E) (*n* = 33), *aac(3)-IId* (*n* = 24), *sul* genes (*n* = 24), *tet*(39) (*n* = 23), and *aph(3′)* genes (*n* = 17).

### 2.3. Plasmid Characteristics of Carbapenem-Resistant Acinetobacter spp.

**Figure 3:**
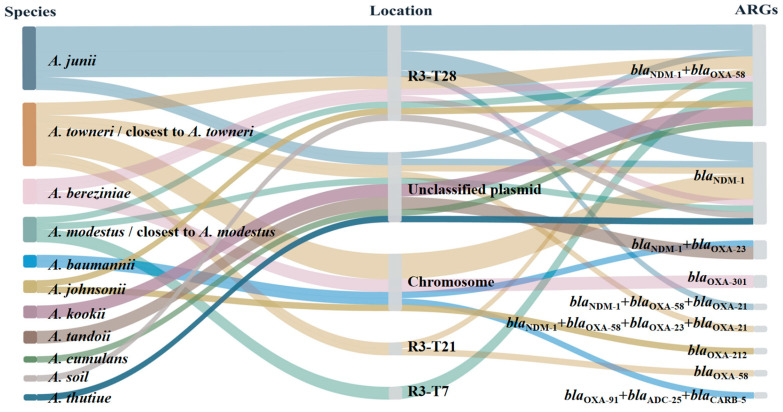
Sankey diagram linking species/group assignment, *bla*_NDM-1_ location, and detected β-lactamase genes in 34 carbapenem-resistant *Acinetobacter* isolates. The nodes “*A. towneri*/closest to *A. towneri*” and “*A. modestus*/closest to *A. modestus*” include both ANI-confirmed isolates and unresolved isolates with highest ANI similarity to the corresponding reference genomes.

To characterize *bla*_NDM-1_ plasmid vehicles, whole-genome sequencing data were used to determine the genomic location of *bla*_NDM-1_, and plasmids were typed using the *Acinetobacter* Plasmid Typing (APT) scheme [29]. The co-occurrence of major carbapenemase genes on *bla*_NDM-1_-positive plasmids was summarized (Figure 3; Table S4). The *bla*_NDM-1_ gene was mainly plasmid-borne (29/34, 85.3%), whereas chromosomal *bla*_NDM-1_ was detected in a minority of isolates (5/34, 14.7%). Co-carriage of additional carbapenemase genes was frequently observed among *bla*_NDM-1_-positive plasmids, most commonly with *bla*_OXA-58_ (18/29, 62.1%).

Among the *bla*_NDM-1_-positive plasmids, 19 belonged to R3-type plasmids (R3-T28, *n* = 15; R3-T21, *n* = 2; R3-T7, *n* = 2), while the remaining 10 plasmids were unclassified with respect to a defined APT replicon type (Figure 3). Among the 29 plasmid-borne *bla*_NDM-1_ isolates, R3-T28 was the most frequent *bla*_NDM-1_ vehicle (15/29, 51.7%) and commonly co-carried *bla*_OXA-58_ (10/15, 66.7%). R3-T21 plasmids were detected in two isolates in the *A. towneri*/closest-to-*A. towneri* group, whereas R3-T7 plasmids were detected in two isolates in the *A. modestus*/closest-to-*A. modestus* group; *bla*_OXA-58_ co-carriage was also observed in these types. In addition, *bla*_OXA-23_ co-carriage with *bla*_NDM-1_ was observed only in two *A. tandoii* isolates (2/34, 5.9%) (Figure 3).

**Figure 4:**
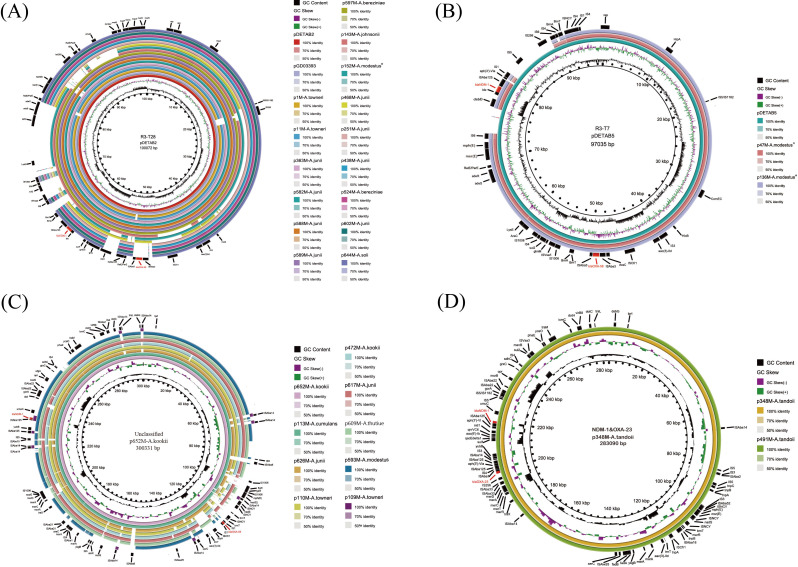
Circular comparisons of representative *bla*_NDM-1_-carrying plasmids. In each panel, a representative plasmid was selected as the reference according to plasmid type and backbone similarity, and related plasmids were aligned as concentric rings. Black and colored inner curves indicate GC content and GC skew, respectively. Ring shading indicates sequence identity (100%, 70%, and 50%) to the reference plasmid. Isolates marked with an asterisk did not reach the 95% ANI species-level cutoff. (**A**) R3-T28 plasmids; (**B**) R3-T7 plasmids; (**C**) Unclassified *bla*_NDM-1_-carrying plasmids; (**D**) Representative *bla*_NDM-1_/*bla*_OXA-23_ co-harboring plasmids from *A. tandoii*.

Among *bla*_NDM-1_-positive plasmids, R3-T28 was the dominant type across *Acinetobacter* spp., and seven R3-T28 plasmids co-harboring *bla*_NDM-1_ and *bla*_OXA-58_ showed high similarity to the reference plasmid pDETAB2 (GenBank: CP047975.1; R3-T28), recovered from a clinical *A. baumannii* isolate in Hangzhou, China, in 2019 and carrying the same two genes (80–99% coverage; 99.43–100% nucleotide identity). The same seven plasmids were also highly similar to the plasmid pGD03393 (GenBank: CP092086.1; R3-T25) from a clinical *A. bereziniae* isolate in Shenzhen, China, in 2022 (94–99% coverage; 99.44–100% nucleotide identity) (Figure 4A). The *bla*_NDM-1_ gene was also located on R3-T7 plasmids in two isolates labeled as closest to *A. modestus*; these plasmids were highly similar to pDETAB5 (GenBank: CP072528.1; R3-T7), which was recovered from a clinical *A. baumannii* isolate in Hangzhou, China, with 82–84% coverage and 99.99–100% nucleotide identity (Figure 4B). In contrast, the two R3-T21 plasmids showed only partial similarity to their respective references pGX5 and pCP038501 (both R3-T21; coverage 44–66%, identity 98.07–99.04%) and carried *bla*_NDM-1_ and/or *bla*_OXA-58_ in IS-associated regions (Figure S2). Ten unclassified *bla*_NDM-1_-carrying plasmids shared conserved backbone blocks but also displayed isolate-specific insertions/deletions across different *Acinetobacter* hosts (Figure 4C). Notably, the two *bla*_NDM-1_/*bla*_OXA-23_ co-harboring plasmids from *A. tandoii* were recovered from two different hospitals in May 2025 and displayed near-identical plasmid backbones in circular comparisons (Figure 4D).

### 2.4. Genetic Environments and Structural Variants of bla_NDM-1_

**Figure 5:**
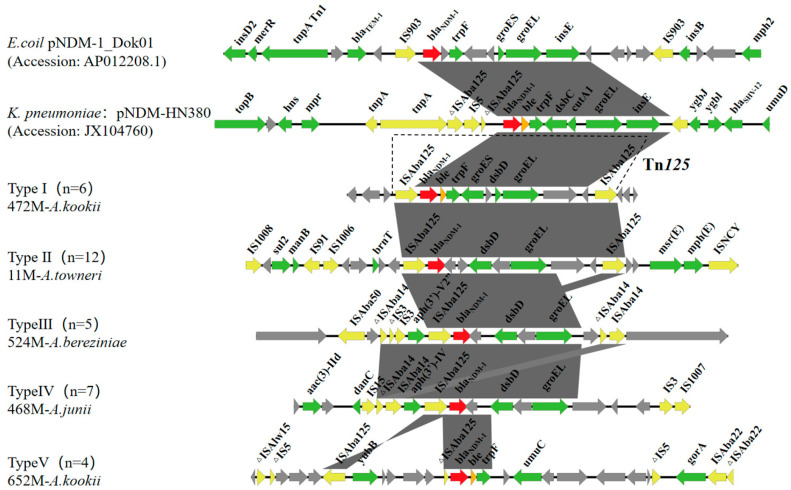
Genetic environments of *bla*_NDM-1_ in representative *Acinetobacter* isolates. Arrows indicate annotated ORFs and transcriptional orientation. Arrow colors denote different ORF categories: red, carbapenemase gene (*bla*_NDM-1_); yellow, insertion sequence (IS)-related genes; green, resistance- or mobility-associated genes; gray, other flanking ORFs. Shaded blocks denote BLASTn (v2.12.0+) nucleotide similarity. The symbol Δ indicates a truncated gene or insertion sequence.

We compared the genetic environments flanking *bla*_NDM-1_ across the 34 isolates using Easyfig (Figure 5). Two *Enterobacterales* plasmids that commonly carry *bla*_NDM-1_ were included for contextual comparison: pNDM-1_Dok01 from a clinical *Escherichia coli* isolate in Japan (GenBank: AP012208.1) and pNDM-HN380 from a hospital-associated *Klebsiella pneumoniae* isolate in Hong Kong (GenBank: JX104760). Despite the multi-species composition of the collection and substantial diversity in the surrounding regions, *bla*_NDM-1_ was consistently embedded in an IS*Aba125*-associated, Tn*125*-related core segment, with IS*Aba125* or truncated IS*Aba125* (ΔIS*Aba125*) present at the boundaries. In contrast, the upstream and downstream regions were altered to varying degrees due to additional inserted sequences (e.g., IS*Aba14*, IS*Aba22*, IS*5*, IS*3*, and IS*1007*). Genes frequently observed in proximity to *bla*_NDM-1_ included *ble*, *trpF*, and the molecular chaperonins *groES*/*groEL*. In a subset of isolates, the *bla*_NDM-1_ region was further linked to other resistance-associated loci, including *msr*(E)/*mph*(E) and *aac(3)-IId*. Overall, these data indicate a conserved IS*Aba125*-linked *bla*_NDM-1_ core embedded within diverse and IS-rich genetic backgrounds in *Acinetobacter*.

## 3. Discussion

This study conducted a one-year, culture-based surveillance of hospital wastewater from five tertiary hospitals in Shenzhen, China, integrating phenotypic susceptibility testing with genomic characterization of carbapenem-resistant *Acinetobacter* isolates. Globally, *bla*_NDM-1_ has been reported most frequently in *Enterobacterales*, particularly *Klebsiella* spp. (52.07%) and *Escherichia* spp. (19.96%), while *Acinetobacter* spp. account for only 11.06% [30]. In contrast, within our selectively cultured *bla*_NDM-1_-positive isolate set, *Acinetobacter* spp. represented 34% (34/100) of the total, suggesting that hospital wastewater may serve as an important reservoir of *bla*_NDM-1_-carrying *Acinetobacter* under the present isolation conditions. This observation is consistent with previous reports from China documenting the prevalence of *bla*_NDM-1_ in *Acinetobacter* spp. across clinical, environmental, and livestock samples [31,32]. The earliest report of an NDM-producing clinical *Acinetobacter* isolate worldwide involved a *bla*_NDM-1_-positive *A. baumannii* strain identified in India [33]. In China, the first documented *bla*_NDM-1_-positive clinical isolates were likewise *A. baumannii*, reported from four provinces in 2010 [22]. However, in our collection, plasmid-borne *bla*_NDM-1_ was most frequently identified in *A. junii* (10/29) and *A. towneri* (6/29), rather than *A. baumannii,* indicating that surveillance focused solely on *A. baumannii* may overlook the potential contribution of NBA to the persistence and dissemination of *bla*_NDM-1_ in wastewater settings.

Notably, *bla*_NDM-1_ was detected in 100% (34/34) of the *Acinetobacter* isolates recovered under meropenem selection. Although *bla*_NDM-1_ prevalence in wastewater-derived *Acinetobacter* varies across settings (e.g., 31.2% in Nigeria and 65% in other Chinese sewage systems) [18,23], every isolate in our collection carried *bla*_NDM-1_, indicating a close association between *bla*_NDM-1_ and carbapenem resistance in this selectively cultured isolate collection. This pattern is consistent with the high regional burden of carbapenemase genes reported from eight teaching hospitals (including Shenzhen) in Guangdong (2022–2024), where carbapenemase-encoding genes were detected in 85.19% (46/54) of carbapenem-resistant *Enterobacter cloacae* isolates [34]. The 100% carriage observed here also exceeds the 55% *bla*_NDM-1_ positivity reported among carbapenem-resistant *Enterobacteriaceae* (CRE) from Indian hospital wastewater [35], further supporting the view that hospital wastewater can act as an important reservoir of *bla*_NDM-1_ under the present sampling and isolation conditions.

Another notable feature of our study was the overwhelming predominance of NBA (33/34), which contrasts with clinical surveillance patterns in which high-risk *A. baumannii* typically dominates hospital-associated infections. Hospital wastewater, however, may represent a broader ecological interface in which *A. baumannii* co-occurs with diverse NBA species [19]. Previous studies from China further suggest that the species composition of *Acinetobacter* in hospital sewage can vary across settings. For example, Gu et al. reported that *A. baumannii* was the predominant *Acinetobacter* species in untreated hospital wastewater from Zhejiang (32/70, 45.7%), which differs from the NBA predominance observed in our collection [19]. In contrast, other studies recovered *bla*_NDM-1_-positive non-*baumannii Acinetobacter*, including *A. johnsonii*, from hospital sewage [27]. Local evidence also indicates that NBA can be recovered in Shenzhen, including *A. tandoii* from a contaminated river [36]. Taken together, these findings suggest that the species composition of *Acinetobacter* in hospital wastewater may vary according to local epidemiology, sampling strategy, and selective culture conditions, and may not directly mirror clinical infection patterns. Against this background, the predominance of NBA in our collection may reflect the ecological diversity of *Acinetobacter* populations under the present sampling and meropenem-selective culture conditions. Because contemporaneous clinical isolates from the participating hospitals were not included in this study, a direct comparison between wastewater species distribution and patient infection patterns could not be performed.

In our study, all *bla*_NDM-1_-positive isolates were resistant to meropenem and exhibited a multidrug-resistant (MDR) phenotype, consistent with the increasing global trend of MDR *A. baumannii* infections [37]. Moreover, our isolates displayed markedly elevated carbapenem MICs (meropenem MIC_50/90_, 32/64 mg/L; imipenem MIC_50/90_, >128/> 128 mg/L), indicating high-level carbapenem resistance. This finding is consistent with CHINET 2024 surveillance, which reported high resistance rates to imipenem (64.5%) and meropenem (64.7%) among clinical *Acinetobacter* isolates in China [38]. Notably, our data highlight a disparity between clinical and wastewater-derived NBA: while clinical cohorts often report sporadic carbapenem resistance among NBA [39,40], our wastewater-derived NBA isolates exhibited uniformly high carbapenem MICs (>32 mg/L), in line with environmental observations that hospital-associated waters can enrich for highly resistant *Acinetobacter* [41]. With the increasing prevalence of carbapenem-resistant Gram-negative bacteria, colistin is increasingly relied upon as a last-resort therapy [42]. In our collection, 21% of isolates were categorized as resistant to colistin, yet no *mcr* genes were detected. This suggests the possible involvement of chromosomal mechanisms such as alterations in the PmrAB two-component system and/or lipid A (LPS) biosynthesis pathways [43]. Overall, these findings highlight hospital wastewater as an under-recognized reservoir of highly resistant MDR *Acinetobacter*, with the potential to maintain and disseminate resistance determinants.

Core-genome SNP analysis revealed several highly related isolate pairs among pre-disinfection hospital sewage samples. These included pairs from the same hospital and season (0 SNP), from different hospitals within the same season (1–2 SNPs), and from the same hospital across seasons (19 SNPs). Such patterns suggest spatio-temporal intermixing of closely related *Acinetobacter* lineages in this collection, potentially reflecting shared upstream inputs and/or recurrent introductions. However, published mutation-rate estimates are derived mainly from clinical *A. baumannii* populations and vary across lineages and analytical settings, and their direct applicability to environmental *Acinetobacter* isolates remains uncertain. Therefore, SNP distances in this study were interpreted conservatively as indicators of close genomic relatedness rather than as direct estimates of transmission time [44,45]. In parallel, the near-ubiquitous presence of *bla*_NDM-1_—typically in Tn*125*-related, IS*Aba125*-associated contexts—and the high prevalence (97.1%) of the *msr*(E)/*mph*(E) macrolide-resistance gene pair across diverse genetic backgrounds suggest the dissemination of conserved resistance units likely associated with mobile genetic elements [10,46]. Taken together, these patterns are compatible with wastewater settings that may favor the maintenance and possible exchange of mobile resistance modules across hosts and niches [47].

The predominant plasmid carriage of *bla*_NDM-1_ (29/34, 85.3%) in our hospital wastewater collection points to a possible role of plasmid-mediated mobility in maintaining carbapenem resistance in this niche. This aligns with global genomic evidence that *bla*_NDM_ is typically plasmid-borne and embedded within transposon-rich, highly recombining regions associated with potential horizontal dissemination [9]. Rep_3 plasmids were the predominant backbones, accounting for 65.5% (19/29) of *bla*_NDM-1_-positive plasmids. This finding mirrors rep-gene-based typing and comparative plasmid surveys identifying Rep_3 as one of the most common and diverse plasmid families in *Acinetobacter* [29,48]. This pattern is also consistent with the species composition of our isolates (33/34 NBA). A recent pan-genus plasmid analysis reported that, in Asia, R3-type AMR plasmids comprise 89.1% of resistance-encoding non-*baumannii Acinetobacter* plasmids, indicating a high regional representation of these plasmid backbones [49]. Within the Rep_3 family, R3-T28 was the most frequent type in our collection (15/19, 78.9%), in line with Tobin et al., who found R3-T28 to be enriched in non-*baumannii Acinetobacter* [49]. While R3-T28 has been reported mainly from clinical datasets, its recovery from hospital sewage here is consistent with ongoing clinical inputs into wastewater. Across species and hospitals, the R3-T28 and R3-T7 plasmids in our collection exhibited highly conserved backbones, with variability largely confined to discrete MGE-rich resistance regions. This architecture mirrors the modular evolution reported for pDETAB2/pDETAB5-related Rep_3 plasmids [50,51]. In addition, the high representation of *A. junii* (8/19, 42.1%) among Rep_3-plasmid carriers indicates that certain NBA lineages may act as important environmental reservoirs at the clinical–wastewater interface. The high sequence identity between our isolates and a clinical *bla*_NDM-1_-positive *A. bereziniae* plasmid from Shenzhen further supports local persistence of these high-risk backbones [52]. Collectively, the repeated recovery of near-identical Rep_3 plasmids across hospitals is consistent with the maintenance of clinically relevant resistance regions within environmental *Acinetobacter* populations in the wastewater setting, with the possibility of further genetic reorganization.

Furthermore, *bla*_OXA-58_ was detected in 18 of these 29 *bla*_NDM-1_-positive plasmids, most often on the R3-T28 backbone (10/18, 55.6%). OXA-58 is a globally distributed class D carbapenemase that has been reported across Europe, Australia, the United States, and Asia [53,54,55,56]. OXA-type carbapenemases (including OXA-58) are also widely established in clinical *Acinetobacter baumannii-calcoaceticus* complex collections [57]. The frequent co-carriage of *bla*_NDM-1_ and *bla*_OXA-58_ on the same plasmid in our dataset (18/29, 62.1%) is consistent with prior reports of plasmid-level co-localization [58,59]. These reports include a clinical *A. pittii* isolate from China and fully resolved MDR plasmids from *A. baumannii* and *A. nosocomialis*. Isolates co-carrying *bla*_NDM-1_ and *bla*_OXA-58_ have also been recovered from hospital sewage (e.g., *A. towneri*), supporting wastewater as an additional niche where such multi-carbapenemase platforms can persist [60]. Much of the published evidence derives from sporadic case reports, which limits inference on how frequently this linkage occurs across species and settings. In comparison, our meropenem-selected yet non-species-targeted recovery from hospital wastewater captured multiple *Acinetobacter* species and revealed frequent plasmid co-carriage of *bla*_NDM-1_/*bla*_OXA-58_ (18/29), thereby providing a broader snapshot beyond clinically curated datasets. Beyond the predominant R3-type plasmids, we identified an emerging pattern in *A. tandoii*: co-carriage of *bla*_NDM-1_ and *bla*_OXA-23_ on highly similar plasmids (Figure 4D). While co-harboring *bla*_NDM-1_ and *bla*_OXA-23_ is a hallmark of high-risk clinical *A. baumannii* clones, its detection in *A. tandoii*—typically associated with aquatic niches—suggests that this resistance configuration may also occur in an environmental NBA species [61,62]. Overall, our findings indicate frequent plasmid co-carriage of *bla*_NDM-1_ with OXA-type carbapenemases in hospital wastewater, with Rep_3/R3-T28 appearing to serve as a major scaffold for the *bla*_NDM-1_-*bla*_OXA-58_ linkage.

To further clarify the genetic contexts associated with carbapenem resistance, we analyzed the flanking regions of *bla*_NDM-1_ and observed a recurrent “conserved core-diverse context” pattern. In all 34 isolates, *bla*_NDM-1_ was immediately associated with IS*Aba125* or ΔIS*Aba125*, consistent with IS*Aba125* being a common signature upstream of *bla*_NDM_ and a likely source of promoter sequences that support expression [9,20,63]. This IS*Aba125*-linked core is consistent with Tn*125*/Tn*125*-derived structures serving as major vehicles for *bla*_NDM-1_ dissemination in *Acinetobacter* and potentially beyond [10,64,65]. In parallel, other carbapenemase loci showed IS-linked organization in our collection (*bla*_OXA-58_ with IS*Aba3*; *bla*_OXA-23_ with IS*Aba1*), and class 1 integron features were detected near *bla*_OXA-23_, suggesting the possibility of co-selection in wastewater [66,67,68,69]. Taken together, our data support the view that hospital wastewater may serve as a setting in which high-risk *bla*_NDM-1_ contexts are repeatedly detected and may undergo further local genetic rearrangement. On this basis, strengthening genomic surveillance for *bla*_NDM-1_ in China remains important, particularly at wastewater-linked One Health interfaces.

## 4. Materials & Methods

### 4.1. Sample Selection and Bacterial Isolates

Wastewater samples from five tertiary hospitals in Shenzhen, China (designated as ET, SY, BD, FY and ZYY), were collected by 24 h composite sampling using an automated sampler (Luban 1A; Shenzhen Wanwu Sensing Technology Co., Ltd., Shenzhen, China) in November 2024 and February, May, and August 2025. Pre- and post-disinfection wastewater samples (3 L each) were transported to the laboratory on ice for immediate processing. Dilutions were prepared at 1:5 and 1:25. Aliquots (100 µL) were inoculated onto Brain Heart Infusion (BHI) agar supplemented with meropenem (2 µg/mL) and vancomycin (30 µg/mL), as well as CHROMagar™ Orientation medium (CHROMagar, Paris, France). Plates were incubated at 37 °C for 18–24 h. For each hospital, 30 colonies representing distinct morphotypes were selected, purified by subculture, and stored for downstream analyses.

### 4.2. Species Identification and Antimicrobial Susceptibility Testing

Genomic DNA was extracted using the boiling method. Nearly full-length 16S rRNA gene fragments were amplified using primers 27F and 1492R, followed by Sanger sequencing. Species identification was performed using BLASTn searches against the NCBI 16S rRNA database [70], and the top hit was used for preliminary taxonomic assignment. To reduce clonal redundancy, all isolates were genotyped using enterobacterial repetitive intergenic consensus PCR (ERIC-PCR) as previously described [71]. MICs were determined by the broth microdilution method according to the Clinical and Laboratory Standards Institute (CLSI) guidelines (CLSI M100, 2025) [72]. Tigecycline MICs were interpreted using the U.S. Food and Drug Administration (FDA) *Enterobacterales* breakpoints. Colistin MICs were determined by broth microdilution and interpreted for *Acinetobacter* spp. as intermediate at ≤2 mg/L and resistant at ≥4 mg/L. *Escherichia coli* ATCC 25922 was included as the quality-control strain in broth microdilution assays. The antimicrobial panel included colistin (CST), gentamicin (GEN), amikacin (AMK), tetracycline (TET), doxycycline (DOX), meropenem (MEM), imipenem (IPM), ciprofloxacin (CIP), cefepime (FEP), ceftazidime (CAZ), tigecycline (TGC), piperacillin/tazobactam (PIP/TAZ), and trimethoprim-sulfamethoxazole (TMP-SMX).

### 4.3. PCR Screening for Carbapenemase Genes

Isolates recovered from meropenem-containing plates were screened by polymerase chain reaction (PCR) for *bla*_NDM_, *bla*_KPC_, and *bla*_OXA-48_-like genes using published primers [73]. PCR products were analyzed by 1.0–1.5% agarose gel electrophoresis and visualized under UV illumination. PCR screening was used as an initial detection step, whereas final confirmation of carbapenemase genes and their genetic contexts was based on whole-genome sequencing rather than amplicon sequencing.

### 4.4. Whole-Genome Sequencing, Assembly, Annotation, and Detection of Resistance and Mobile Elements

Genomic DNA for sequencing was extracted using the TIANGEN Wizard Genomic DNA Kit (Tiangen Biotech, Beijing, China). DNA concentration was quantified using a Qubit 3.0 fluorometer with the Qubit dsDNA HS Assay Kit (Thermo Fisher Scientific, Waltham, MA, USA). Short-read libraries were sequenced using the MGI DNBSEQ-G99 platform, and long-read sequencing was performed using the Oxford Nanopore MinION platform. After adapter trimming and quality filtering, hybrid assemblies were generated using Unicycler v0.4.6 [74]. Genome annotation was performed using Bakta v1.11 [75]. Preliminary species identification was first inferred by 16S rRNA sequencing. Whole-genome-based taxonomic identification was further supported using PubMLST [76]. In addition, ANI analysis against reference genomes was used as the explicit criterion for final species assignment. Isolates with ANI values ≥95% were considered confidently assigned at the species level, whereas isolates with ANI values below this cutoff were conservatively treated as unresolved at the species level. ARGs were detected using ResFinder v2.1, and MGEs, including insertion sequences and secretion system-related features, were annotated using VRprofile2 [77]. Plasmid replicon typing for *Acinetobacter* spp. was performed using the APT scheme based on replication initiation (rep) genes [29]. Web BLAST searches were performed using default parameters against the NCBI nucleotide database. Comparative plasmid analyses were conducted by aligning representative plasmids to publicly available references retrieved from NCBI GenBank, including pDETAB2 (CP047975.1), pGD03393 (CP092086.1), pDETAB5 (CP072528.1), pGX5 (CP071769.1), and *Acinetobacter baumannii* strain CIAT758 plasmid unnamed1 (CP038501.1). Visualization tools included Easyfig v2.2.3 [78] and BRIG v0.95 for circular plasmid comparisons [79], TBtools-II v2.376 for heatmap generation [80], and iTOL v7 for phylogenetic trees visualization [81].

## 5. Conclusions

This study combined a one-year, culture-based investigation of wastewater collected from five tertiary hospitals in Shenzhen with AST and whole-genome sequencing of carbapenem-resistant *Acinetobacter*. The *bla*_NDM-1_ gene was detected in all carbapenem-resistant *Acinetobacter* isolates and was predominantly plasmid-borne, with the Rep_3 family, particularly R3-T28, acting as a major vehicle that frequently co-harbored *bla*_OXA-58_. We also identified an emerging threat in *A. tandoii* isolates carrying *bla*_NDM-1_/*bla*_OXA-23_ plasmids, highlighting the capture of clinically relevant resistance determinants by environmental hosts. The modular organization of these Tn*125*-like structures further suggests that IS-driven diversification as a key force structuring the wastewater resistome. The predominance of non-*baumannii Acinetobacter* in this collection likely reflects the broader ecological diversity of wastewater-associated *Acinetobacter* under the present sampling and meropenem-selective culture conditions.

A limitation of this study is the restriction of sampling to discrete time points within a single city, which may limit inferences regarding finer temporal dynamics and broader geographic patterns. Nevertheless, our data support hospital wastewater as an important reservoir for high-risk carbapenemase determinants. These data advocate for the routine integration of hospital wastewater monitoring into “One Health” surveillance and emphasize the urgent need for targeted source-control strategies to mitigate the environmental dissemination of carbapenem resistance.

## Figures and Tables

**Table 1:** Antimicrobial resistance rates of 34 carbapenem-resistant *Acinetobacter* spp. isolates against 13 antimicrobial agents.

Antibiotic	MIC Range (mg/L)	MIC _50_(mg/L)	MIC _90_(mg/L)	Resistance (%)
Cefepime	0.002–128	128	>128	94
Ceftazidime	0.002–128	>128	>128	100
Piperacillin/Tazobactam	0.25/4–128/4	128/4	128/4	53
Meropenem	0.002–128	32	64	100
Imipenem	0.002–128	>128	>128	100
Amikacin	0.25–128	2	16	9
Gentamicin	0.006–128	128	>128	68
Tetracycline	0.25–128	8	32	47
Doxycycline	0.25–128	0.25	2	3
Ciprofloxacin	0.002–128	8	64	67
Colistin	0.006–128	2	8	21
Tigecycline	0.002–128	0.25	1	0
Trimethoprim-Sulfamethoxazole	0.002/0.04–128/2432	32/608	>128/2432	76

## Data Availability

Genome assemblies have been deposited in the China National Center for Bioinformation (CNCB) Genome Warehouse (GWH) under BioProject PRJCA052902 and are publicly released.

## Supplementary Materials

The following supporting information can be downloaded at: https://www.mdpi.com/article/10.3390/antibiotics15040347/s1. Figure S1. Distribution of 100 bacterial strains with *bla*_NDM_-positive genes. Figure S2. Circular alignments of reference plasmid sequences with homologous contigs from carbapenem-resistant *Acinetobacter* spp. recovered in this study. (A) R3-T21 plasmid. (B) R3-T21 plasmid. Table S1. ANI-based species assignment of 34 *Acinetobacter* isolates using reference genomes. Table S2. The MIC values of 34 carbapenem-resistant *Acinetobacter* spp. Isolates in antimicrobial susceptibility testing. Table S3. Pairwise core-genome SNP distances among carbapenem-resistant *Acinetobacter* isolates. Table S4. Distribution and genetic characteristics of NDM-1-carrying carbapenem-resistant *Acinetobacter* isolates identified in this study.
