# Supplementary material for: Genomic Epidemiology of NDM-1 Carbapenemase-Producing Acinetobacter spp. from Hospital Wastewater in Shenzhen, China
Source: Antibiotics (Basel). 2026 Mar 27;15(4):347. doi: 10.3390/antibiotics15040347 (PMC13113592; doi:10.3390/antibiotics15040347)
Supplement: Supplementary file 1 [file antibiotics-15-00347-s001.zip › Supplementary Materials.pdf]

Genomic Epidemiology of NDM-1 Carbapenemase-Producing *Acinetobacter* spp. from Hospital Wastewater in Shenzhen, China

Xiaoqian Guo [1,2,†], Yulin Fu [2,†], Xinxin Chen [3], Yiying Cheng [1], Huimin Li [2], Dalin Hu [1], Suli Huang [4], Liangqiang Lin [2,*] and Ziquan Lv [1,2,*]

[1] School of Public Health, Southern Medical University, Guangzhou 510515, China; xq1214201@163.com (X.G.); cyy0066882024@163.com (Y.C.); smuhdl@126.com (D.H.)
[2] Division of Conservation and Application of Biological Resources, Shenzhen Center for Disease Control and Prevention, Shenzhen 518055, China; fyl199287@163.com (Y.F.); lihuimin0203@126.com (H.L.)
[3] School of Medicine, Southern University of Science and Technology, Shenzhen 518055, China; 12333058@mail.sustech.edu.cn
[4] School of Public Health, Shenzhen University Medical School, Shenzhen University, Shenzhen 518055, China; grace420@szu.edu.cn
* Correspondence: szlinqiang6@163.com (L.L.); lvziquan1984@126.com (Z.L.)
† These authors contributed equally to this work.

.

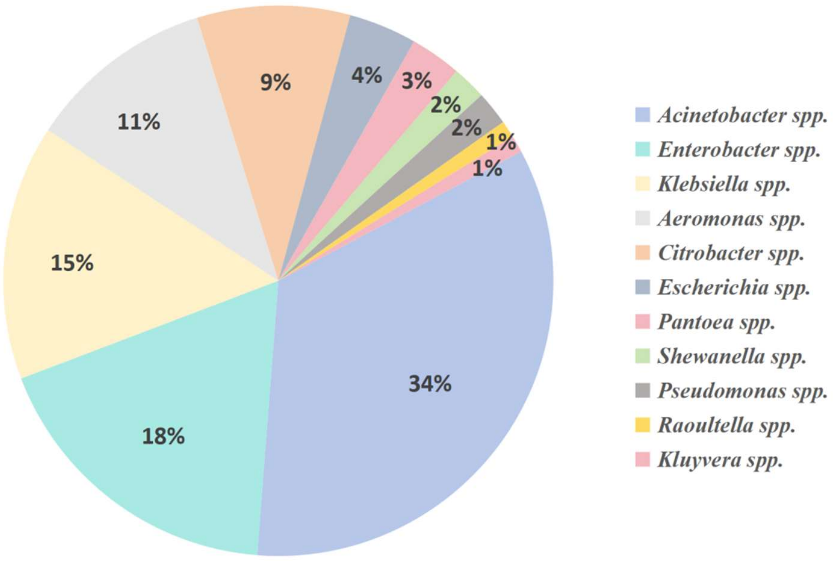

Figure S1. Distribution of 100 bacterial strains with *bla*NDM-positive genes.

Table S1. ANI-based species assignment of 34 Acinetobacter isolates using reference genomes

Isolate	Preliminary identification	Final assignment by ANI	Closest reference accession	ANI (% identity)
1M	*A.towneri*	*A. towneri*	GCF_000368785.1	97.556
11M	*A.towneri*	*A. towneri*	GCF_000368785.1	97.5522
40M	*A.towneri*	*Acinetobacter* sp. (closest to *A. towneri*)	GCF_017498585.1	94.0329
47M	*A.modestus*	*Acinetobacter* sp. (closest to *A. modestus*)	GCF_000367965.1	94.8214
81M	*A.towneri*	*Acinetobacter* sp. (closest to *A. towneri*)	GCF_017498585.1	93.9577
109M	*A.towneri*	*A. towneri*	GCF_000368785.1	97.284
110M	*A.towneri*	*A. towneri*	GCF_000368785.1	97.6901
113M	*A.cumulans*	*A. cumulans*	GCF_003024525.3	97.8161
136M	*A.modestus*	*Acinetobacter* sp. (closest to *A. modestus*)	GCF_000367965.1	94.8084
143M	*A.johnsonii*	*A. johnsonii*	GCF_900444855.1	95.8988
152M	*A.modestus*	*Acinetobacter* sp. (closest to *A. modestus*)	GCF_014636095.1	94.7484
251M	*A.junii*	*A. junii*	GCF_900444865.1	97.9815
290M	*A.baumannii*	*A. baumannii*	GCF_009035845.1	97.7069
342M	*A.towneri*	*Acinetobacter* sp. (closest to *A. towneri*)	GCF_017498585.1	93.8782
348M	*A.tandoii*	*A. tandoii*	GCF_000400735.1	96.9265
363M	*A.junii*	*A. junii*	GCF_900444865.1	97.4286
425M	*A.towneri*	*A. towneri*	GCF_000368785.1	97.2244
438M	*A.junii*	*A. junii*	GCF_900444865.1	98.0807
459M	*A.towneri*	*Acinetobacter* sp. (closest to *A. towneri*)	GCF_017498585.1	93.9874
468M	*A.junii*	*A. junii*	GCF_900444865.1	97.3158
472M	*A.kookii*	*A.kookii*	GCF_039543765.1	97.3117
491M	*A.tandoii*	*A. tandoii*	GCF_000400735.1	96.978
524M	*A.bereziniae*	*A. bereziniae*	GCF_000368925.1	97.8831

582M	*A.junii*	*A. junii*	GCF_900444865.1	98.1725
588M	*A.junii*	*A. junii*	GCF_900444865.1	97.8355
589M	*A.junii*	*A. junii*	GCF_900444865.1	98.258
593M	*A.modestus*	*A. modestus*	GCF_014636095.1	96.289
597M	*A.bereziniae*	*A. bereziniae*	GCF_000368925.1	97.8526
602M	*A.junii*	*A. junii*	GCF_900444865.1	97.9699
609M	*A.sp.*	*A. thutiue*	GCF_030385805.1	96.2945
617M	*A.junii*	*A. junii*	GCF_900444865.1	97.9306
626M	*A.junii*	*A. junii*	GCF_900444865.1	97.2603
644M	*A.soli*	*A. soli*	GCF_000760595.1	98.4508
652M	*A.kookii*	*A. kookii*	GCF_039543765.1	97.1615

Note: ANI ≥95% was considered supportive of species-level assignment. Isolates with ANI <95% were conservatively reported as Acinetobacter sp. and annotated according to their closest reference genome.

Table S2. The MIC values of 34 carbapenem-resistant *Acinetobacter* spp. Isolates in antimicrobial susceptibility testing.

Isolate	Antibiotics												
	CST	GEN	AMK	TET	DOX	MEM	IPM	CIP	FEP	CAZ	TGC	PIP/TAZ	TMP-SMX
1M	1	>128	0.5	16	2	64	>128	16	128	>128	0.25	128/4	64/1216
11M	0.5	>128	0.5	64	2	64	>128	8	>128	>128	2	>128/4	>128/2432
40M	2	>128	128	32	1	64	>128	32	64	>128	0.25	128/4	64/1216
47M	32	>128	1	64	0.5	64	>128	>128	>128	>128	0.25	>128/4	>128/2432
81M	0.5	0.5	1	16	1	16	>128	8	32	>128	1	16/4	0.5/9.5
109M	2	1	16	>128	64	16	>128	4	128	>128	0.25	32/4	128/2432
110M	2	1	8	32	2	16	>128	1	32	>128	0.25	128/4	64/1216
113M	2	2	0.5	0.25	0.25	32	>128	>128	128	>128	0.25	128/4	4/76
136M	2	128	0.5	0.5	0.25	64	>128	2	>128	>128	0.25	128/4	>128/2432
143M	0.25	0.25	1	1	2	32	>128	4	>128	>128	0.25	64/4	16/304
152M	16	>128	4	1	0.25	64	>128	8	128	>128	0.25	64/4	1/19
251M	4	>128	4	8	0.5	8	32	0.25	8	>128	0.25	0.25/4	128/2432
290M	2	4	16	1	0.25	64	>128	32	>128	>128	0.25	>128/4	64/1216
342M	1	4	1	8	0.25	8	32	32	32	>128	0.25	0.5/4	>128/2432
348M	2	>128	8	16	0.25	16	>128	16	64	>128	0.25	128/4	64/1216
363M	1	>128	1	8	0.25	32	>128	8	128	>128	0.25	64/4	128/2432
425M	2	16	2	8	0.5	32	128	2	128	>128	1	128/4	64/1216
438M	4	>128	8	16	0.5	32	>128	32	64	>128	1	64/4	8/152
459M	2	0.5	16	4	0.25	16	128	32	32	>128	0.25	32/4	2/38
468M	2	32	128	0.5	0.25	64	>128	>128	128	>128	0.25	128/4	0.5/9.5
472M	1	0.5	0.25	16	0.5	8	32	0.25	64	>128	0.25	32/4	32/608

491M	2	128	8	16	0.25	16	128	16	64	>128	0.25	128/4	32/608
524M	8	>128	16	8	0.25	128	>128	2	>128	>128	0.25	>128/4	0.5/9.5
582M	1	>128	1	8	0.5	64	128	1	128	>128	0.25	>128/4	4/76
588M	0.5	128	1	16	2	32	128	0.25	128	>128	2	>128/4	16/304
589M	1	128	1	16	0.5	64	>128	1	128	>128	0.25	64/4	8/152
593M	4	4	4	16	0.5	32	128	8	32	>128	0.25	8/4	0.5/9.5
597M	16	128	0.5	8	0.25	128	>128	2	64	>128	0.25	128/4	128/2432
602M	1	>128	8	8	0.25	64	>128	32	128	>128	0.25	64/4	64/1216
609M	1	>128	1	4	0.25	64	>128	8	32	>128	0.25	8/4	8/152
617M	1	0.5	2	1	0.25	64	>128	1	16	>128	0.25	4/4	0.5/9.5
626M	1	16	128	1	0.25	32	>128	16	32	>128	0.25	16/4	0.5/9.5
644M	1	>128	16	16	0.25	128	>128	4	>128	>128	0.5	>128/4	4/76
652M	1	64	2	32	1	64	>128	64	>128	>128	2	>128/4	64/1216

Abbreviations: CST, colistin; GEN, gentamicin; AMK, amikacin; TET, tetracycline; DOX, doxycycline; MEM, meropenem; IPM, imipenem; CIP, ciprofloxacin; FEP, cefepime; CAZ, ceftazidime; TGC, tigecycline; PIP/TAZ, piperacillin/tazobactam; TMP-SMX, trimethoprim-sulfamethoxazole.

Table S4. Distribution and genetic characteristics of NDM-1-carrying carbapenem-resistant *Acinetobacter* isolates identified in this study.

Isolate	Year	Hospital	Species	Plasmid -type	β-lactamases ARGs	
					on plasmid	on chromosome
1M	2024	ETP	*A. towneri*	R3-T28	*bla*$_{\text{NDM-1}}$、*bla*$_{\text{OXA-58}}$	–
11M	2024	ETP	*A. towneri*	R3-T28	*bla*$_{\text{NDM-1}}$、*bla*$_{\text{OXA-58}}$	–
342M	2025	ETP	*A. towneri**	–	–	*bla*$_{\text{NDM-1}}$
348M	2025	ETP	*A.tandoii*	Unassigned	*bla*$_{\text{NDM-1}}$、*bla*$_{\text{OXA-23}}$	–
459M	2025	ETP	*A. towneri**	–	–	*bla*$_{\text{NDM-1}}$
524M	2025	ETP	*A. bereziniae*	R3-T28	*bla*$_{\text{NDM-1}}$	*bla*$_{\text{OXA-301}}$
644M	2025	ETP	*A. soli*	R3-T28	*bla*$_{\text{NDM-1}}$	–
40M	2024	SYP	*A. towneri**	–	–	*bla*$_{\text{NDM-1}}$
290M	2025	SYP	*A. baumannii*	–	–	*bla*$_{\text{NDM-1}}$、*bla*$_{\text{OXA-23}}$、*bla*$_{\text{OXA-91}}$、*bla*$_{\text{ADC-25}}$、*bla*$_{\text{CARB-5}}$
363M	2025	SYP	*A. junii*	R3-T28	*bla*$_{\text{NDM-1}}$、*bla*$_{\text{OXA-58}}$、*bla*$_{\text{OXA-21}}$	–
468M	2025	SYP	*A. junii*	R3-T28	*bla*$_{\text{NDM-1}}$、*bla*$_{\text{OXA-58}}$	–
47M	2024	BDP	*A. modestus**	R3-T7	*bla*$_{\text{NDM-1}}$、*bla*$_{\text{OXA-58}}$	–
136M	2024	BDP	*A. modestus**	R3-T7	*bla*$_{\text{NDM-1}}$、*bla*$_{\text{OXA-58}}$	–
472M	2025	BDP	*A. kookii*	Unassigned	*bla*$_{\text{NDM-1}}$、*bla*$_{\text{OXA-58}}$	–
652M	2025	BDP	*A. kookii*	Unassigned	*bla*$_{\text{NDM-1}}$、*bla*$_{\text{OXA-58}}$	–
81M	2024	FYP	*A. towneri**	–	–	*bla*$_{\text{NDM-1}}$
143M	2024	FYP	*A. johnsonii*	R3-T28	*bla*$_{\text{NDM-1}}$、*bla*$_{\text{OXA-58}}$	*bla*$_{\text{OXA-212}}$
251M	2025	FYP	*A. junii*	R3-T28	*bla*$_{\text{NDM-1}}$	–
425M	2025	FYP	*A. towneri*	R3-T21	*bla*$_{\text{NDM-1}}$、*bla*$_{\text{OXA-58}}$	–
582M	2025	FYP	*A. junii*	R3-T28	*bla*$_{\text{NDM-1}}$、*bla*$_{\text{OXA-58}}$	–
588M	2025	FYP	*A. junii*	R3-T28	*bla*$_{\text{NDM-1}}$、*bla*$_{\text{OXA-58}}$	–
589M	2025	FYP	*A. junii*	R3-T28	*bla*$_{\text{NDM-1}}$、*bla*$_{\text{OXA-58}}$	–
593M	2025	FYP	*A. modestus*	Unassigned	*bla*$_{\text{NDM-1}}$	–
597M	2025	FYP	*A. bereziniae*	R3-T28	*bla*$_{\text{NDM-1}}$、*bla*$_{\text{OXA-58}}$	*bla*$_{\text{OXA-301}}$
602M	2025	FYP	*A. junii*	R3-T28	*bla*$_{\text{NDM-1}}$	–
109M	2024	ZYYP	*A. towneri*	R3-T21	*bla*$_{\text{NDM-1}}$、*bla*$_{\text{OXA-58}}$	–
110M	2024	ZYYP	*A. towneri*	Unassigned	*bla*$_{\text{NDM-1}}$、*bla*$_{\text{OXA-58}}$、*bla*$_{\text{OXA-21}}$、*bla*$_{\text{OXA-23}}$	–
113M	2024	ZYYP	*A. cumulans*	Unassigned	*bla*$_{\text{NDM-1}}$、*bla*$_{\text{OXA-58}}$	–
152M	2024	ZYYP	*A. modestus**	R3-T28	*bla*$_{\text{NDM-1}}$、*bla*$_{\text{OXA-58}}$	–
438M	2025	ZYYP	*A. junii*	R3-T28	*bla*$_{\text{NDM-1}}$	–
491M	2025	ZYYP	*A. tandoii*	Unassigned	*bla*$_{\text{NDM-1}}$、*bla*$_{\text{OXA-23}}$	–
609M	2025	ZYYP	*A. thutiue*	Unassigned	*bla*$_{\text{NDM-1}}$	–
617M	2025	ZYYP	*A. junii*	Unassigned	*bla*$_{\text{NDM-1}}$	–
626M	2025	ZYYP	*A. junii*	Unassigned	*bla*$_{\text{NDM-1}}$、*bla*$_{\text{OXA-58}}$	–

Abbreviations: ARGs, antibiotic resistance genes.

Note: Isolates marked with an asterisk (*) did not reach the ANI cutoff of 95% for

confident species-level assignment. These isolates were annotated according to their closest related species. Detailed ANI-based species assignments are provided in Table S1.

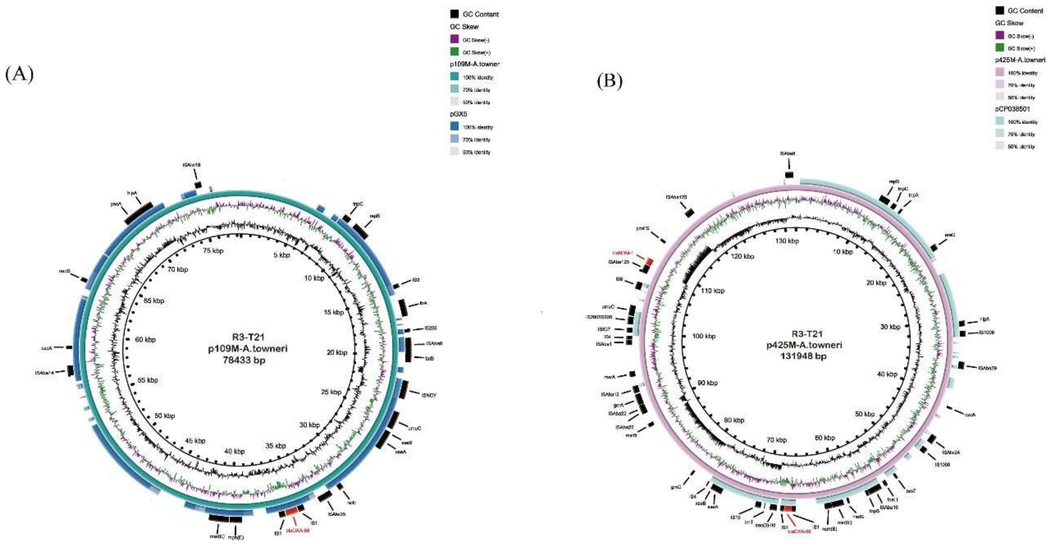

Figure S2. Circular alignments of reference plasmid sequences with homologous contigs from carbapenem-resistant *Acinetobacter* spp. recovered in this study. (A) R3-T21 plasmid. (B) R3-T21 plasmid.
